# Investigation of microwave application time with constant pulse ratio on drying of zucchini

**DOI:** 10.1002/fsn3.3458

**Published:** 2023-05-22

**Authors:** Jalal Dehghannya, Sepideh Farhoudi, Saeed Dadashi

**Affiliations:** ^1^ Department of Food Science and Technology University of Tabriz Tabriz Iran

**Keywords:** drying, energy consumption, intermittent microwave, low temperature, microstructure

## Abstract

Since nonuniform drying in the continuous microwave harms the safety and quality of dried food materials, a significant quality improvement can be achieved by controlling the application time of the microwave. This study aimed to investigate the influence of microwave application time and power at a constant pulse ratio during the drying of zucchini on different product characteristics. The samples were first exposed to microwaves with powers of 360, 600, and 900 W alternately for 10, 30, and 50 s. After drying with intermittent microwaves, the process was continued using the hot‐air drying. Increasing the microwave application time and power caused a significant reduction in the total process time. The minimum drying time was obtained at 900 W and 50 s (259.44 min). D_eff_ increased significantly by 24.4% and 34.1% with increasing application time and power, respectively. The highest shrinkage and bulk density were observed in the samples dried at 360 W and 10 s due to a longer total process time than the other treatments. Rehydration increased by 10.3% and 14.7% with increasing application time and power, respectively. A 33% decrease in energy consumption was noticed in the 900 W–50 s treatment compared to the 360 W–10 s treatment. Moreover, with increasing microwave application time and power, the lightness of the dried product decreased, and the total color difference increased. In summary, the 900 W power and 50 s application time produced a better‐dried product than the other treatments considering different quantitative and qualitative properties. The results of this research can be used in the food industry to dry products using microwave and hot air to control and improve their quality.

## Graphical abstract

At constant pulse ratio, microwave application time (MAT) was controlled to dry zucchini. Moisture diffusivity enhanced by 24.4% by increasing the MAT, lowering drying time. The rehydration ratio increased by 10.3% as the MAT increased from 10 to 50 s. A 33% decrease in energy consumption was observed in the 900 W power and 50 s MAT. Pulsed microwave using suitable time with hot air is suggested for industrial drying applications.

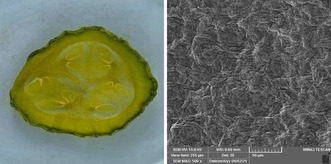

## 1 INTRODUCTION

Zucchini with the scientific name *Cucurbita pepo* L. belongs to the *Cucurbits* family. This product has low calories (Bagheri & Dinani, 2019) and contains a suitable level of B vitamins and minerals (Chayjan et al., 2017). Drying is an old way of preserving food for longer storage by avoiding the growth of microorganisms (Chayjan et al., 2017). Dried zucchini is a functional food with high magnitudes of fiber and bioactive compounds (Soquetta et al., 2018) that can be used in seasoning and soup mixtures.

Hot‐air convective drying is considered the most common drying technique due to its simple control (Bantle & Eikevik, 2014). However, usually, the drying rate by convection is low, the drying time is long, and as a result, its energy consumption is high. In this method, a significant increase in temperature causes the loss of nutrients and the reduction of product quality characteristics such as color (Arikan et al., 2012; Rodríguez et al., 2018). To solve the problems of hot‐air drying, using microwave energy with proper control can be a fast, safe, and straightforward method (Khan et al., 2020). Heat is generated by microwaves with dipole rotation and ionic polarization. Changes in the electric field cause the rotation of bipolar (water) molecules, the movement of ions in food, and as a result, creation of friction and volumetric heating (Chahbani et al., 2018; Kumar & Karim, 2019). Among the advantages of microwave drying compared to the convection method, higher drying rate, lower energy consumption, lower shrinkage, and higher rehydration ratio can be mentioned (Wray & Ramaswamy, 2015).

However, continuous use of microwave energy during the drying process causes a drop in the product's quality characteristics due to the creation of hot and cold spots attributed to the interference of electromagnetic waves in the microwave chamber. This causes nonuniformity of temperature and moisture profiles, and as a result, the drying process is performed non‐uniformly (Pitchai et al., 2012; Vadivambal & Jayas, 2010). To solve this problem, the microwave can be used intermittently. Application of alternating or pulsed microwave energy due to a uniform distribution of heat and moisture inside the product, when the microwave is “off,” prevents irregular heating and enhances the quality of the product.

When the food is exposed to microwave energy, a lot of heat is generated, and it causes moisture to be quickly removed from the product's texture (Dehghannya, Bozorghi, & Heshmati, 2018). To enhance the performance of microwave drying, this method is applied simultaneously with other drying techniques such as convection, freeze, vacuum, fluidized bed, and osmotic dehydration. Since methods such as freeze drying and vacuum drying involve a lot of capital and operating costs, the convective drying method is primarily used in combination with microwaves. The problems related to the convective form, including the long drying time and the formation of a hard crust on the product, can be significantly reduced in the combined approach due to enhancing the diffusion rate of moisture from the inside to the surface of the food and the provision of sufficient surface moisture. Combining the microwave method with convection can reduce drying time, increase product quality and save energy. In the convection method, the air temperature has a significant effect on the evaporation rate, drying rate, and process time. Increasing the drying temperature due to the increase in the effective moisture diffusion coefficient (D_eff_) causes a rapid removal of water and, consequently, the reduction of the processing time. However, the quick removal of moisture leads to the creation of tension inside and on the surface of the product, and samples that have been dried at a higher temperature suffer more shrinkage and less rehydration capacity; Therefore, the lower the drying air temperature, the better the product quality in terms of structure and rehydration (Chayjan et al., 2015; Jia et al., 2019). On the other hand, using high temperatures for a long time leads to severe damage to the color, taste, and overall quality of heat‐sensitive products (Das & Arora, 2018).

Based on the available information, so far, no research has been reported investigating the effect of microwave application time with constant pulse ratio in combined pulsed–hot‐air drying of zucchini at a low temperature (40°C). The current research aimed to investigate the simultaneous effect of microwave powers and application times in a joint drying process on the quantitative and qualitative characteristics of the product.

## 2 MATERIALS AND METHODS

### 2.1 Raw material

Zucchini (*Cucurbita pepo* L.) was purchased from a local market and stored in a cold room with a temperature of 4°C before conducting the experiments (Eissa et al., 2013). The initial moisture content of zucchini based on dry mass was 21.215 ± 1.476 g water/g dry solids. Toluene was used to measure the volume of samples by the liquid displacement method.

### 2.2 Preparation of samples

The samples were cut using a mold and a cutter to a diameter of 6 cm and a thickness of 5 mm. Blanching of the samples was carried out in a hot water bath equipped with a temperature sensor (Bain‐marie model WM22, FanAzma Gostar Company), with a sample to water ratio of 1–5 at 90°C for 2 min (Paciulli et al., 2020). After blanching, moisture absorbent paper was used to remove excess moisture from the surface of the samples.

### 2.3 Drying

A combined microwave–hot‐air dryer (SolarDOM‐LG model SD‐3855 SCR, with internal dimensions of 480 × 392 × 527 mm, the capacity of 38 L, South Korea) with the ability to adjust the microwave power in levels 90, 180, 360, 600, and 900 W at a frequency of 2450 MHz equipped with an air temperature adjustment system in the range of 40–230°C and also a rotating tray with a rotation speed of 2.5 rpm. First, the samples were exposed to microwaves with powers of 360, 600, and 900 W (1.11, 1.85, and 2.78 W/g) alternately for periods of 10, 30, and 50 s (microwaves' application times) with a fixed pulse ratio of 4.

The pulse ratio was expressed using the following relationship (Soysal et al., 2009):

(1) $$\mathrm{PR}=\frac{t_{\mathrm{on}}+t_{\mathrm{off}}}{t_{\mathrm{on}}}$$

where *t*_on_ is the time when the microwave is “on” (applying the waves), and *t*_off_ is the time when the microwave is “off” in s.

**FIGURE 1:**
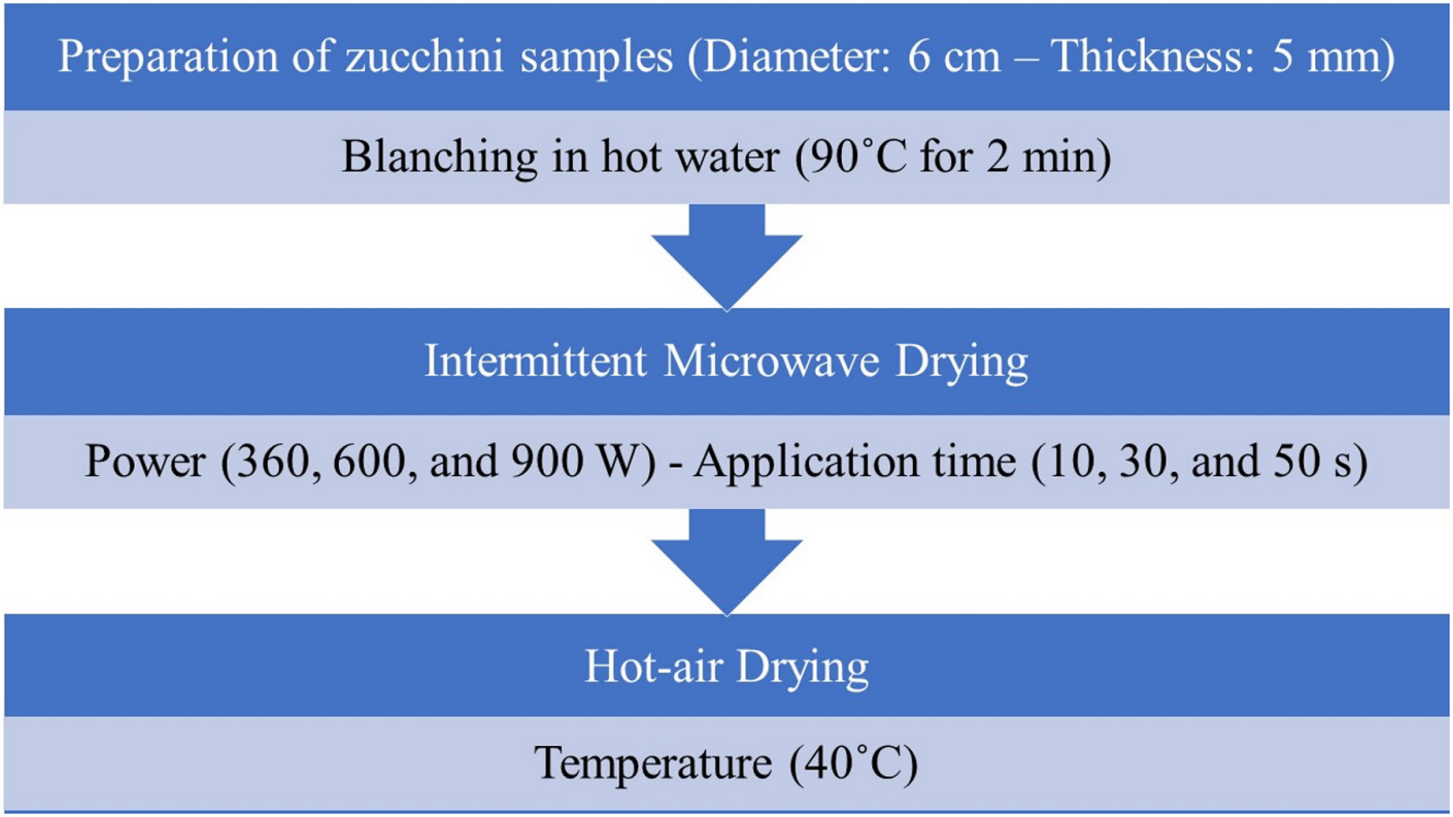
Flowchart of the combined intermittent microwave–hot‐air drying of zucchini slices.

The microwaves' application times were determined through trial and error so that the pulsed microwave heating was stopped before the product started to burn. After drying with pulsed microwaves, the process was continued using the hot‐air convection method at a temperature of 40°C until the moisture content of the samples reached 0.2 g water/g dry solids (Chayjan et al., 2017; Dehghannya, Hosseinlar, & Heshmati, 2018). The “on” and “off” times of the microwave in different application times and powers were according to Table 1. A flowchart of the combined intermittent microwave–hot‐air drying of zucchini slices is shown in Figure 1.

**TABLE 1:** Microwave on/off times at powers of 360, 600, and 900 W and constant pulse ratio of 4.

Microwave “on” time (s)	Microwave “off” time (s)	Microwave total time (“on” + “off”) (s)
10	30	40
30	90	120
50	150	200

### 2.4 Moisture content (MC)

The zucchini samples were placed in an oven (model BM120, FanAzma Gostar Company, Iran, capacity 120 L, smart and equipped with an air circulation fan) at 105°C under atmospheric pressure 24 h until a constant mass was reached. The MC on a dry basis (d.b.) was calculated according to the following equation (Dehghannya, Hosseinlar, & Heshmati, 2018):

(2) $$\mathrm{MC}\left(d.b.\right)=\frac{M_{w}}{M_{S}}$$

where *M*_W_ is the mass of water (g), and *M*_S_ is the mass of dry solids in the sample (g).

### 2.5 Drying rate (DR)

The drying rate was calculated according to the following equation (Bagheri & Dinani, 2019):

(3) $$\mathrm{DR}=\frac{M_{t}-M_{t+\mathrm{\Delta t}}}{\Delta t}$$

where DR is drying rate (g water/g dry solids. min), *t* is time (min), Δ*t* is time difference (min), and *M*_t + Δt_ and *M*_t_ are the moisture content at time *t* + Δ*t* and *t* (g water/g dry solids), respectively.

### 2.6 Effective moisture diffusion coefficient (*D*_eff_)

Since moisture diffusion is considered the dominant mechanism of moisture transfer from inside to the surface of the food during drying, the moisture ratio was calculated based on Fick's second law to describe unsteady moisture diffusion by the Crank's equation for an infinite slab assuming uniform initial moisture distribution (Dehghannya, Bozorghi, & Heshmati, 2018):

(4) $$\mathrm{MR}=\frac{M_{t}-M_{e}}{M_{0}-M_{e}}=\frac{8}{\pi^{2}}\sum_{n=1}^{\infty }\frac{1}{{\left(2n-1\right)}^{2}}\exp\left(-\frac{{\left(2n-1\right)}^{2}\pi^{2}D_{\mathrm{eff}}}{4L^{2}}\right)t$$

where MR is the moisture ratio, *M*_t_ is the moisture content at the time *t* (g water/g dry matter), *M*_0_ and *M*_e_ are, respectively, the initial moisture and equilibrium moisture (g water/g dry matter), *D*_eff_ is the effective moisture diffusion coefficient (m^2^/s), *L* is half the thickness of the sample (m), and *t* is the drying time (s).

For long periods of drying, only the first term of the series is considered, and equation 3 becomes:

(5) $$\mathrm{MR}=\frac{M_{t}}{M_{0}}=\frac{8}{\pi^{2}}\;\exp\left(\frac{-\pi^{2}D_{\mathrm{eff}}}{4L^{2}}t\right)$$

By taking the logarithm of both sides of equations 5 and 6 is obtained:

(6) $$\mathrm{Ln}\left(\mathrm{MR}\right)=\ln\left(\frac{8}{\pi^{2}}\right)-\left(\frac{\pi^{2}D_{\mathrm{eff}}}{4L^{2}}\right)t$$

*D*_eff_ was obtained by plotting the logarithm of moisture ratio against time and then calculating the slope of equation 6:

(7) $$\text{Slope}=\frac{-\pi^{2}}{4L^{2}}D_{\mathrm{eff}}$$

### 2.7 Shrinkage

In general, shrinkage indicates volume changes and was calculated by the following equation (Dehghannya et al., 2016a):

(8) $$S=\left(1-\frac{V_{t}}{V_{0}}\right)\times 100$$

where *S* is the percentage of shrinkage, *V*_t_ is the apparent volume of the dried sample after time t (cm^3^), and *V*_0_ is the apparent volume of the sample before drying (cm^3^). To measure the apparent volume, the liquid displacement method was applied using a glass pycnometer. First, the pycnometer was filled entirely with toluene and weighed. Then, the samples were transferred into the pycnometer half filled with toluene, and the remaining volume of the pycnometer was filled with toluene, and its mass was determined. The apparent volume (*V*) was calculated according to the following equations:

(9) $$V=V_{f}-\frac{M_{\mathrm{sf}}}{\rho_{s}}$$

(10) $$M_{\mathrm{sf}}=M_{t+s}-M_{f}-M$$

where *V*_f_ is the volume of the flask (cm^3^), *M*_sf_ is the mass of toluene added to fill the flask (g), *M*_t + s_ is the mass of the flask plus the mass of the toluene and the sample (g), *M*_f_ is the mass of the flask (g), *M* is the mass of the sample (g), and ρ_s_ is the density of toluene (0.87 g/cm^3^ at 20°C).

### 2.8 Bulk density

The bulk density was determined based on the ratio of sample mass to its volume (Dehghannya et al., 2016b):

(11) $$\rho_{b}=\frac{m_{t}}{V_{t}}$$

where ρ_b_ is bulk density (g/cm^3^), *m*_t_ is the mass of the sample (g), and *V*_t_ is the volume of the sample (cm^3^) at time t.

### 2.9 Rehydration ratio

Twenty grams of the dried sample was immersed in 100 mL of distilled water at 50°C inside a beaker in a bain‐marie for 60 min. Then, the samples were removed from the water, and the excess water was slowly dried using a moisture absorbent paper. Finally, after weighing the samples, the rehydration ratio was calculated using the following equation (Bagheri & Dinani, 2019):

(12) $$R\left(\%\right)=\frac{M_{t}-M_{0}}{M_{0}}\times 100$$

where *M*_0_ is the mass of the dried sample (g) before rehydration and *M*_t_ is the mass of the dried sample (g) after rehydration.

### 2.10 Microstructure

To investigate the effect of microwave power and application time on the microstructure of dried zucchini slices, a scanning electron microscope (Model MIRA3 FEG‐SEM, Tescan, Czech Republic) with a voltage of 15 kV and a magnification of 500 was used. Imaging was done after covering the samples with a thin layer of gold (Bagheri & Dinani, 2019).

### 2.11 Specific energy consumption

The specific energy consumed in the combined pulsed microwave–hot‐air drying was calculated according to the following equations (Dehghannya, Bozorghi, & Heshmati, 2018):

(13) $$E=E_{1}+E_{2}$$

where *E*_1_ is the specific energy consumption of microwaves (J/kg) and *E*_2_ is the specific energy consumption of hot air (J/kg). *E*_1_ and *E*_2_ were calculated as follows:

(14) $$E_{1}=\frac{Pt_{m}}{\mathrm{PR}\times m_{1}}$$

where *P* is the microwave power (W), *t*_m_ is the drying time of the sample in the microwave (s), PR is the pulse ratio, and *m*_1_ is the moisture content removed during microwave drying (kg).

(15) $$E_{2}=\frac{AV_{a}\rho_{a}∆Ht_{c}}{m_{2}}$$

where *A* is the dryer area containing the sample (m^2^), *V*_a_ is the air velocity (m/s), ρ_a_ is the air density (kg/m^3^), Δ*H* is the air enthalpy (J/kg), t_c_ is the time of drying with hot air (s), and *m*_2_ is the moisture content removed during drying with hot air (kg).

The ΔH is determined as:

(16) $$∆H=\left(C_{p.a}+WC_{p.v}\right)\left(T_{\text{in}}-T_{\mathrm{amb}}\right)+\mathrm{w\lambda}$$

where *C*_p.a_ is the specific heat capacity of air (J/kg °C), *W* is the absolute air humidity (kg water/kg dry air), *C*_p.v_ is the specific heat capacity of water vapor (J/kg °C), *T*_in_ is the internal temperature of the dryer (°C), *T*_amb_ is the ambient temperature (°C), and λ is the latent heat of evaporation (J/kg).

### 2.12 Color

To measure the color of dried zucchinis, first, imaging was done from the dried samples under suitable light. The lighting system included two lamps on both sides of a white frame with a trapezoidal cross‐section at a distance of 30 cm from the sample with an angle of 45 degrees. Then, using Image j software, the obtained RGB factors were converted to L*, a*, and b*. These parameters include lightness or L* from black (0) to white (100), redness or a* from green (negative values) to red (positive values), and yellowness or b* from blue (negative values) to yellow (positive values). The overall color change (ΔE), which indicates the amount of color change of the sample after drying compared to the initial sample, was obtained from the following relationship (Dehghannya et al., 2017):

(17) $$\Delta E=\sqrt{{\left(L_{0}-L^{*}\right)}^{2}+{\left(a_{0}-a^{*}\right)}^{2}+{\left(b_{0}-b^{*}\right)}^{2}}$$

where *L*_0_, *a*_0_, and *b*_0_ are color parameters before drying and L*, a*, and b* are color parameters after drying.

The chroma index, which indicates the degree of saturation or color intensity, was obtained based on the following equation:

(18) $$\text{choroma}={\left(a^{2}+b^{2}\right)}^{\frac{1}{2}}$$

Hue angle was obtained using:

(19) $$\mathrm{hue}\;\text{Angle}=\tan^{-1}\left(\frac{b}{a}\right)$$

where 0 and 360 degrees indicate red color, 90, 180, and 270 degrees indicate yellow, green, and blue colors, respectively.

The browning index, as one of the other indicators of food color, was obtained through the following relationship:

(20) $$\mathrm{BI}=\frac{\left(100-\left(X-0.31\right)\right)}{0.71}$$

where *X* is obtained using:

(21) $$X=\frac{a+1.75L}{5.645L+a-3.012b}$$

### 2.13 Statistical analysis

Statistical analysis was executed using SAS software (version 9.4) as a 3 × 3 × 3 factorial experiment (microwave power at three levels of 360, 600, and 900 W, microwave application time at three levels of 10, 30, and 50 s with three repetitions) in the form of a completely randomized design to study the variables used, including drying kinetics, drying rate, effective moisture diffusion coefficient, shrinkage, bulk density, rehydration, specific energy consumption, and color during drying zucchini slices. The mean comparison was analyzed based on Duncan's multi‐range test at the 5% probability level (*p* < .05).

## 3 RESULTS AND DISCUSSION

### 3.1 Drying kinetics

**FIGURE 2:**
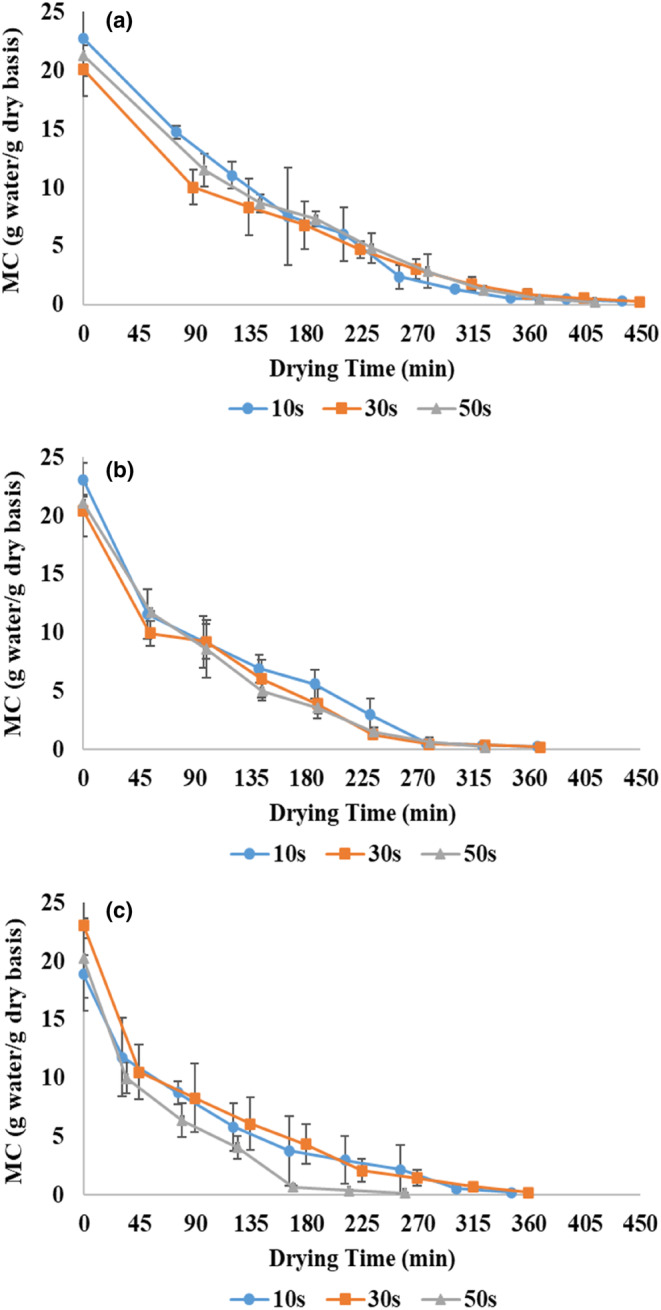
Drying kinetics of zucchini slices at 360 (a), 600 (b), and 900 W (c) microwave powers and application times of 10, 30, and 50 s.

Drying kinetics show the variations of moisture content over drying time. Figure 2a–c shows the drying kinetics of zucchini using pulsed microwaves and hot air at different powers and application times. At all the powers used, the moisture removal rate in the first stage (drying with microwave) was higher (steeper slope) than in the second stage (drying with hot air). In Figure 2a–c, the second marker indicates the completion of the first stage and the beginning of the second stage. The use of microwaves in the first drying stage causes volumetric heating inside the sample, and the rapid release of water vapor leads to porosity development and rapid water evaporation (Dehghannya, Bozorghi, & Heshmati, 2018). At the start of the drying, due to the high initial moisture content of the samples, the bipolar molecules (water) are high. As a result, the absorption of electromagnetic energy by the food increases, and the absorbed energy is converted into thermal energy (Aghilinategh et al., 2015). Following this, the water near the product's surface, the free water, as well as the water that has a weak bond, are easily removed (Maskan, 2001). As the drying process continued (the second step using hot air), the moisture content decreased at a slower rate; Because with the passage of drying time in all treatments, the moisture level of the product decreased, and the resistance to moisture removal increased (Salahi et al., 2015).

**FIGURE 3:**
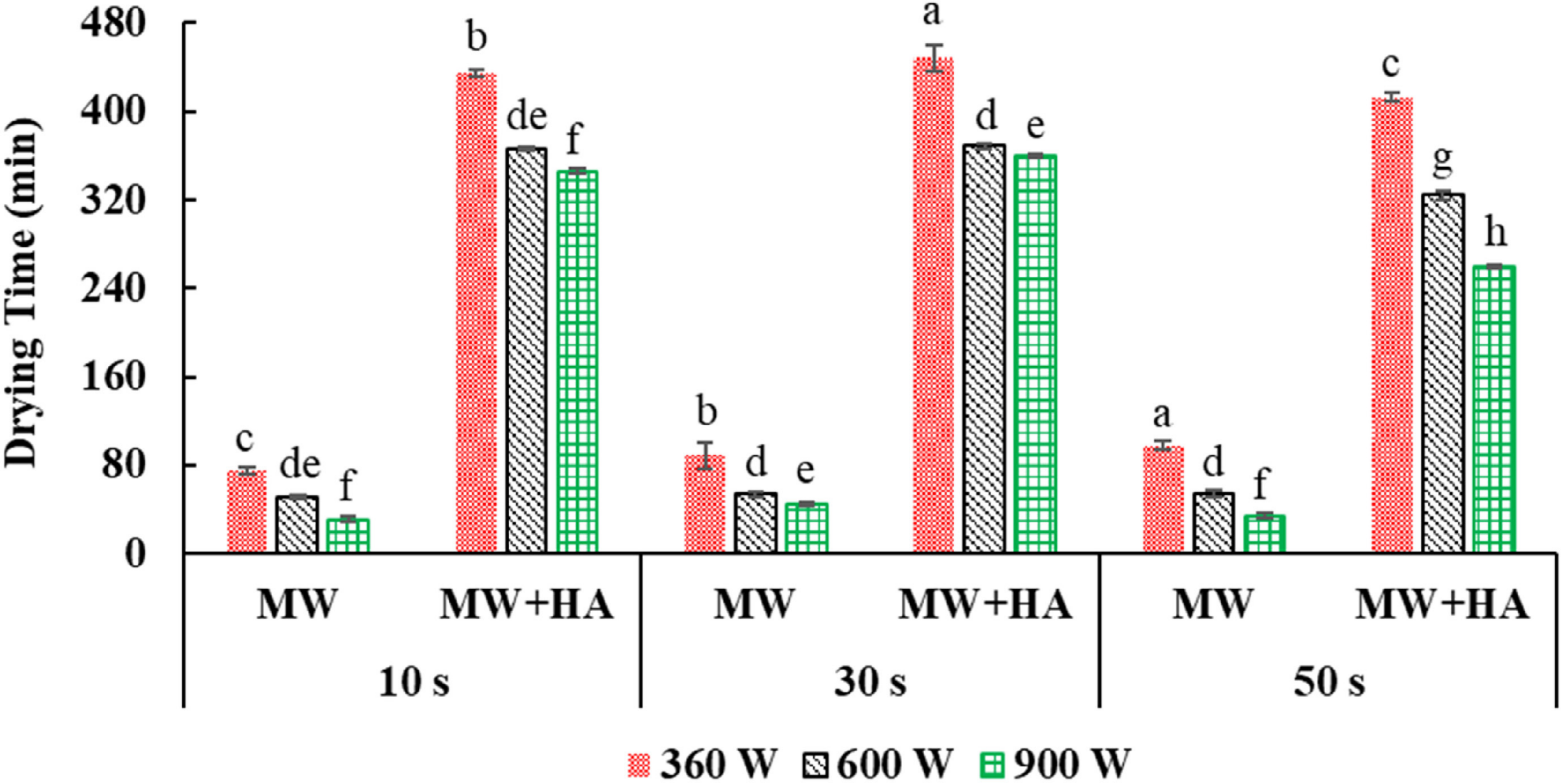
Drying time after microwave drying (MW), and total drying time [microwave and hot‐air drying (MW + HA)] taking into account microwave “on” and “off” times. Different letters for the same drying operation (MW or MW + HA) indicate a significant difference (*p* < .05).

**FIGURE 4:**
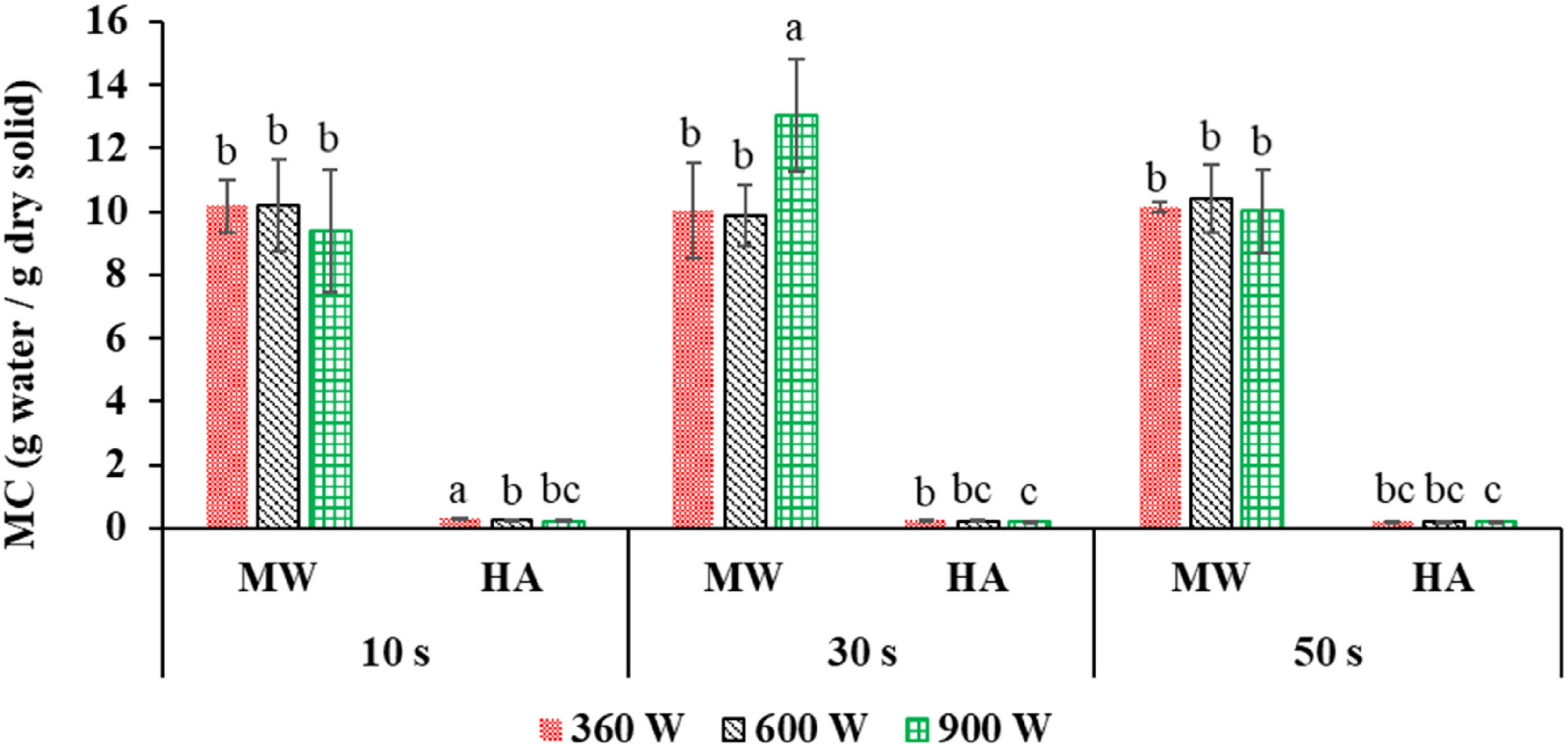
Moisture content (MC) after microwave drying (MW) and after hot‐air drying (HA) at 360, 600, and 900 W microwave powers and application times of 10, 30, and 50 s. Different letters for the same drying operation (MW or HA) indicate a significant difference (*p* < .05).

In general, the microwave drying time increased by increasing the microwave application time from 10 to 50 s (Figure 3). Because of the constant pulse ratio, microwave “off” time increases with increasing application time (Table 1), and the sample experiences more temperature drop (in the shutdown phase). Therefore, generally, the moisture content at the end of the first stage (after microwave application) increased with increasing application time and lengthening the microwave cycle (Figure 4). Similar results were obtained by Xu et al. (2017). On the other hand, the time of the whole process (combination of microwave and hot air) decreased by increasing the application time from 10 to 50 s (considering all the applied powers) on average by 13.1% (Figure 3), and the final moisture content (after applying hot air) also decreased (Figure 4). This was related to the increased shutdown time at higher application times at the constant pulse ratio (Table 1). Because in longer “off” times due to the uniform distribution of heat and moisture, the removal of water from the inside to the surface of the product is facilitated, and in the next stage (hot air), the removal of moisture continues more intensively (Dai et al., 2019). This result was in line with the findings of Dai et al. (2019) that by increasing the application time from 4 to 7 s, the total drying time reached from 3 to 1.5 h. This indicated the effect of shutdown time on the drying rate.

In addition, by increasing the power, the drying time with microwaves decreased (Figure 3). This was attributed to the production of more heat inside the product and the increase in the mass transfer rate at higher powers, which caused the rapid removal of moisture from the samples (Sharma & Prasad, 2004). The moisture content in different powers at the end of microwave drying did not generally change (Figure 3); In other words, the time to reach an almost identical moisture content was shorter at higher power (Figure 3). On the other hand, the time of the whole process (combination of microwave and hot air) decreased significantly (on average by 25.5%) by increasing the power from 360 to 900 W (considering all three application times) (Figure 3), and the final moisture content (after applying hot air) also decreased considerably (Figure 4). This can be attributed to reducing the exposure time of the samples to microwaves (Figure 3), reducing the damage to the tissue, and removing more moisture in the subsequent drying stage (hot air). These findings were in agreement with the results of Dai et al. (2019), Tepe and Tepe (2020), and Dehghannya et al. (2021).

Figure 4 shows that by considering the mutual effect of microwave power and application time, the highest and lowest final moisture content was obtained in 360 W power–10 s application time and 900 W power–50 s application time, respectively. Because by increasing the power and application time simultaneously, the activity intensity of water molecules increases, and more heat is produced. This increase in temperature causes internal vapor pressure in the sample, and after that, evaporation occurs faster and more moisture is removed from the sample. In addition, simultaneously increasing the microwave power and application time led to a decrease in the total drying time so that the treatments dried at 900 W–application time 50 s and 360 W–application time 10 s had the shortest and longest drying time, respectively (Figure 3). This is related to the high volumetric heating produced and the increase in the moisture removal rate. In a similar study, Dai et al. (2019) showed that the drying time dropped by enhancing the power and time of microwave application in each cycle.

### 3.2 Drying rate

**FIGURE 5:**
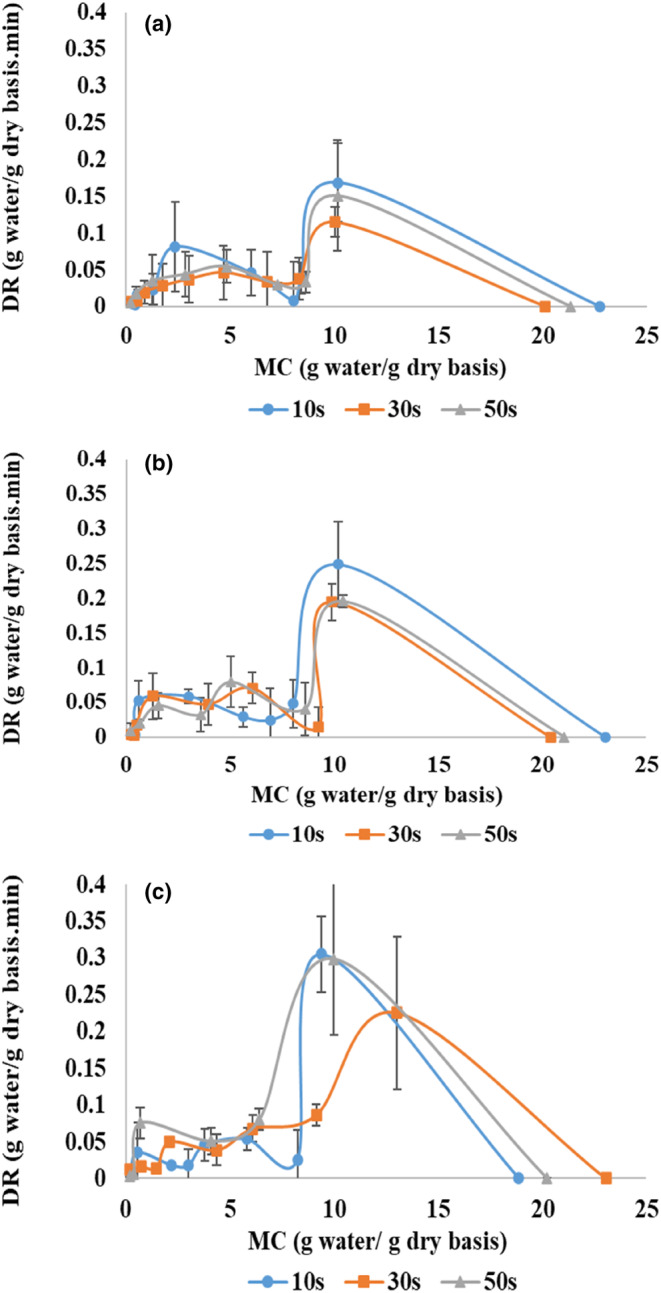
Drying rate of zucchini slices at 360 (a), 600 (b), and 900 W (c) microwave powers and application times of 10, 30, and 50 s.

Figure 5a–c shows the drying rate of zucchini using the pulsed microwave–hot‐air method at different powers and application times. At all treatments in the first drying stage (pulsed microwave), the drying rate increased rapidly. The second marker from the right indicates the completion of the first stage and the beginning of the second stage (hot air). At the start of the drying, due to the high content of moisture and free water in the product, as well as the dipolar nature of water molecules, more heat is generated, and the drying rate increases (Huang et al., 2021). At the start of the second drying stage, a significant drop in the drying rate was observed (starting drying with hot air). This was due to the interruption of electromagnetic waves and the break of volumetric heating production. As the process continued, the moisture on the surface of the samples was gradually removed by hot air. The drying curve decreased with a relatively mild slope in the final stages of the drying; because, over time, and with the moisture removal from the surface of the sample, the resistance to moisture transfer increases (Dehghannya et al., 2019).

**FIGURE 6:**
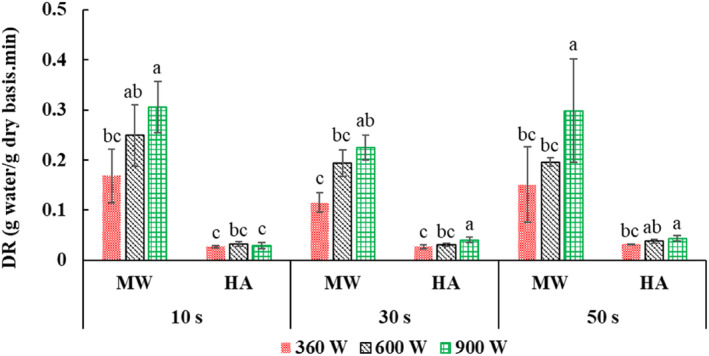
Drying rate (DR) after microwave drying (MW) and after hot‐air drying (HA) at 360, 600, and 900 W microwave powers and application times of 10, 30. and 50 s. Different letters for the same drying operation (MW or HA) indicate a significant difference (*p* < .05).

The drying rate in the first stage (MW) decreased with increasing microwave application time from 10 to 30 s (Figure 5). However, in terms of the application time of 50 s, a nonsignificant increase was observed. This result can be attributed to the increase in shutdown time by increasing the time of microwave application (Table 1) and the general growth in the drying time with microwave (Figure 3). These results were consistent with the findings of Huang et al. (2021), who showed that the drying rate curve became less steep as the shutdown time increased. In addition, the drying rate (hot‐air stage) increased substantially with microwave application time from 10 to 50 s (Figure 6). This increase is probably due to the effect of increasing the application time in transferring more moisture from the inside to the surface of the samples (Figure 4), expanding the shutdown time and its positive effect on the uniform distribution of moisture and heat, and, as a result, better removal of water in the hot‐air phase.

On the other hand, Figure 6 shows that the drying rate increased significantly by increasing the microwave power in the first drying stage (pulsed microwave) (Figure 6). This was related to the reduction of microwave drying time (Figure 3). These findings were in agreement with the results of Dai et al. (2019), Arslan et al. (2020), and Huang et al. (2021). Also, the drying rate (hot‐air phase) increased with increasing power (Figure 6). This result is related to the positive effect of increasing power in creating extensive heating, creating pores, and facilitating water removal in the hot‐air stage, and reducing the total drying time (Figure 3; Dehghannya et al., 2021).

In general, the highest drying rate after the microwave stage was obtained in the dried samples at 900 W power–10 s time (Figure 6). This was due to the release of a certain amount of moisture in a shorter period than other treatments (Figure 3). Also, the highest drying rate in the hot‐air stage was obtained in the dried samples at 900 W–50 s (Figure 6). With the increase in power and application time, due to the increase in volumetric heating in the product and the increase in shutdown time (Table 1), the distribution of moisture, and heat in the product becomes more uniform, and more water is removed from the sample in a shorter time (Figure 3).

### 3.3 Effective moisture diffusion coefficient (*D*_eff_)

**FIGURE 7:**
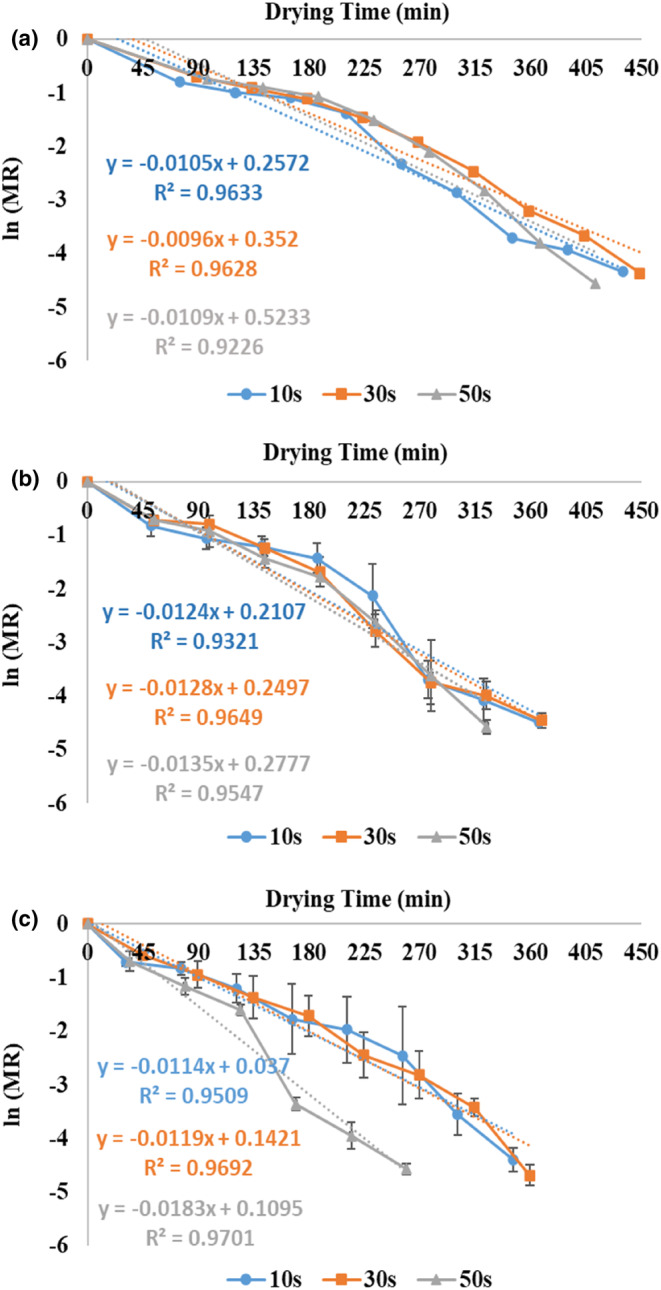
Ln (MR) against drying time of zucchini slices at 360 (a), 600 (b), and 900 W (c) microwave powers and application times of 10, 30, and 50 s.

In Figure 7a–c, the values of ln (MR) against the drying time of zucchini at powers of 360, 600, and 900 W and application times of 10, 30, and 50 s are shown. In general, the slope of ln (MR) versus drying time followed a downward trend in all treatments, and in the equation of all the curves, the slope was attained with a negative sign. The negative slope indicated the decrease in the moisture content of the samples over time (Figure 2).

In general, the *D*_eff_ increased by 24.4% on average as the time of microwave application increased from 10 to 50 s considering all applied powers (Table 2). With the increase in microwave application time, microwave drying time and shutdown time increased (Table 1 and Figure 3). This helped to distribute heat and moisture evenly and facilitated the removal of water in the hot‐air stage, increasing *D*_eff_ and finally reducing the total process time (Figure 3). This finding was consistent with the results of Dai et al. (2019).

**TABLE 2:** Effective moisture diffusion coefficient (*D*_eff_; m^2^/s), shrinkage (Sh; %), bulk density (BD; g/cm^3^), and rehydration ratio (RR; %) of zucchini slices as influenced by different microwave application times and powers.

Microwave application time (s)	Microwave power (W)	*D*_eff_	Sh	BD	RR
10	360	2.651 × 10^−8ef^ ± 1.896 × 10^−9^	60.833^a^ ± 2.231	0.080^a^ ± 0.002	279.21^d^ ± 10.698
10	600	2.442 × 10^−8f^ ± 8.376 × 10^−10^	58.848^ab^ ± 3.310	0.079^a^ ± 0.010	325.25^c^ ± 8.725
10	900	2.758 × 10^−8def^ ± 5.796 × 10^−10^	56.031^bcd^ ± 3.379	0.075^ab^ ± 0.005	333.59^bc^ ± 27.213
30	360	3.148 × 10^−8bc^ ± 1.418 × 10^−9^	56.265^bc^ ± 1.039	0.074^ab^ ± 0.005	332.30^bc^ ± 3.593
30	600	3.243 × 10^−8bc^ ± 1.934 × 10^−9^	56.397^bc^ ± 1.965	0.072^ab^ ± 0.009	331.68^bc^ ± 11.015
30	900	3.422 × 10^−8b^ ± 3.535 × 10^−9^	55.081^bcd^ ± 0.509	0.070^ab^ ± 0.003	349.20^abc^ ± 21.121
50	360	2.890 × 10^−8cde^ ± 2.896 × 10^−9^	54.866^bcd^ ± 1.555	0.072^ab^ ± 0.002	345.06^abc^ ± 19.889
50	600	3.015 × 10^−8cd^ ± 3.288 × 10^−10^	53.012^cd^ ± 3.244	0.065^b^ ± 0.008	358.21^ab^ ± 30.168
50	900	4.629 × 10^−8a^ ± 1.318 × 10^−9^	51.894^d^ ± 0.533	0.068^ab^ ± 0.002	373.11^a^ ± 3.554

*Note*: Different letters in the same column indicate a significant difference (*p* < .05).

Also, the *D*_eff_ increased on average by 34.1% with microwave power from 360 to 900 W considering all application times (Table 2). This result can be related to the rise in the movement of dipolar water molecules and their collisions with increasing power, creating more volumetric heating, increasing the internal vapor pressure difference, and facilitating the transfer of moisture to the surface of the sample (Figure 4; Dehghannya, Bozorghi, & Heshmati, 2018). These results were in line with the findings of Dai et al. (2019), Tepe and Tepe (2020), Arslan et al. (2020), and Dehghannya et al. (2021).

Table 2 shows the *D*_eff_ in drying zucchini by pulsed microwave–hot‐air method at different powers and application times. The highest and lowest *D*_eff_ were, respectively, related to the dried samples at 900 W–50 s and the dried treatments at 360 W–30 s. The simultaneous increase in the microwave power and application time causes faster heating of the product. Then the creation of higher internal vapor pressure leads to the ability to diffuse more moisture from the inside to the surface of the sample (Sharma & Prasad, 2004).

### 3.4 Shrinkage

**FIGURE 8:**
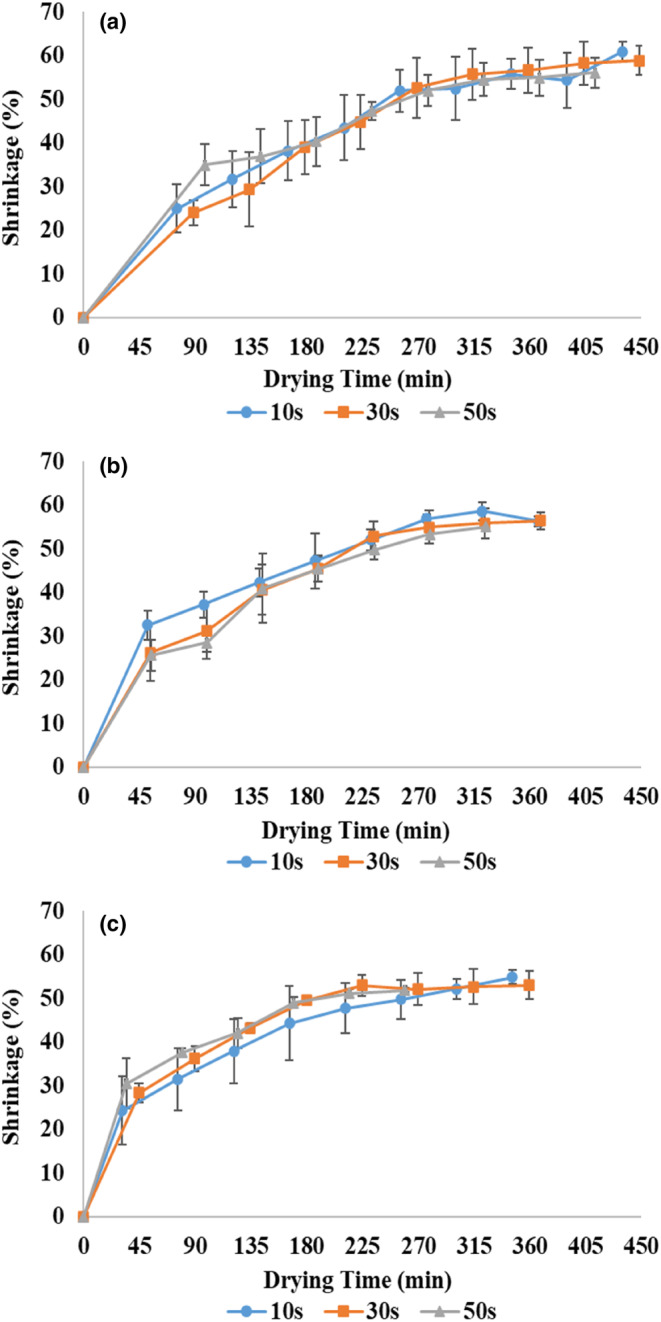
Shrinkage of zucchini slices at 360 (a), 600 (b), and 900 W (c) microwave powers and application times of 10, 30, and 50 s.

Figure 8a–c shows the shrinkage against the drying time of zucchini samples using the pulsed microwave–hot‐air method at different powers and the application times. The shrinkage in all samples increased over drying time; Because over time, the removal of moisture leads to a drop in the volume of samples and an increase in shrinkage. Until the end of the first drying stage (the second marker from the left), when the product was dried using pulsed microwaves, the shrinkage increased steeply. This result can be attributed to the high moisture content at the beginning of the process, which removes a lot of moisture from the product using microwaves. By the end of the process (drying with hot air), the difference between two consecutive shrinkages (slope) decreased. This can be related to the decrease in moisture content, the phenomenon of surface hardening, and the stabilization of the volume of the samples in the final drying stage (Dehghannya et al., 2016a).

In general, the results showed that the final shrinkage of the product decreased on average by 5.2% by increasing the microwave application time from 10 to 50 s considering all applied powers (Table 2). As the time of microwave application increased, *D*_eff_ increased (Table 2), and more moisture was removed from the samples. As a result, the shrinkage of the product decreased by reducing the total drying time (Figure 3). On the other hand, with the increase in the microwave application time at a fixed pulse ratio, the shutdown time also increased (Table 1), and the sample had enough time to adjust the temperature and moisture. Reducing the internal temperature and moisture gradient of the product, in addition to reducing the internal stress, leads to a reduction in drying time and, as a result, a reduction in shrinkage (Aghilinategh et al., 2015; Dehghannya, Bozorghi, & Heshmati, 2018).

In addition, the shrinkage of the product was reduced 9% on average by increasing the power from 360 to 900 W considering all application times (Table 2). This finding was consistent with the results of Maskan (2001). The reduction in the shrinkage was related to the drop in drying time using microwaves (Figure 3) and the decline of the total drying time (Figure 3) with increasing power. This was attributed to the production of extensive heat with increased power (Maskan, 2000), increased moisture diffusion from the inside to the surface of the product, and acceleration of moisture removal (Horuz & Maskan, 2015) and, as a result, increased porosity during the process (Dehghannya et al., 2021).

In addition, the results showed that the maximum shrinkage was related to the dried samples at the 360 W–10 s (Table 2). Because the total drying time in the lower power and time of the microwave increased due to the low moisture removal rate (Figure 3) and caused more shrinkage (Horuz & Maskan, 2015). On the other hand, due to the shorter time of applying the waves in the fixed pulse ratio, the shutdown time is also shorter (Table 1). As a result, the product suffers more stress due to insufficient time to distribute heat and moisture evenly (Pham et al., 2018). Also, the slightest shrinkage was related to the samples dried at 900 W–50 s. Applying high power and time simultaneously increases the internal vapor pressure, develops more pores in the product and quickly removes moisture, and reduces shrinkage (Aghilinategh et al., 2015; Horuz et al., 2017). Horuz and Maskan (2015) attributed the reduction of the processing time and shrinkage due to the use of high powers to the rapid drying of the product's surface compared to the center.

### 3.5 Bulk density

**FIGURE 9:**
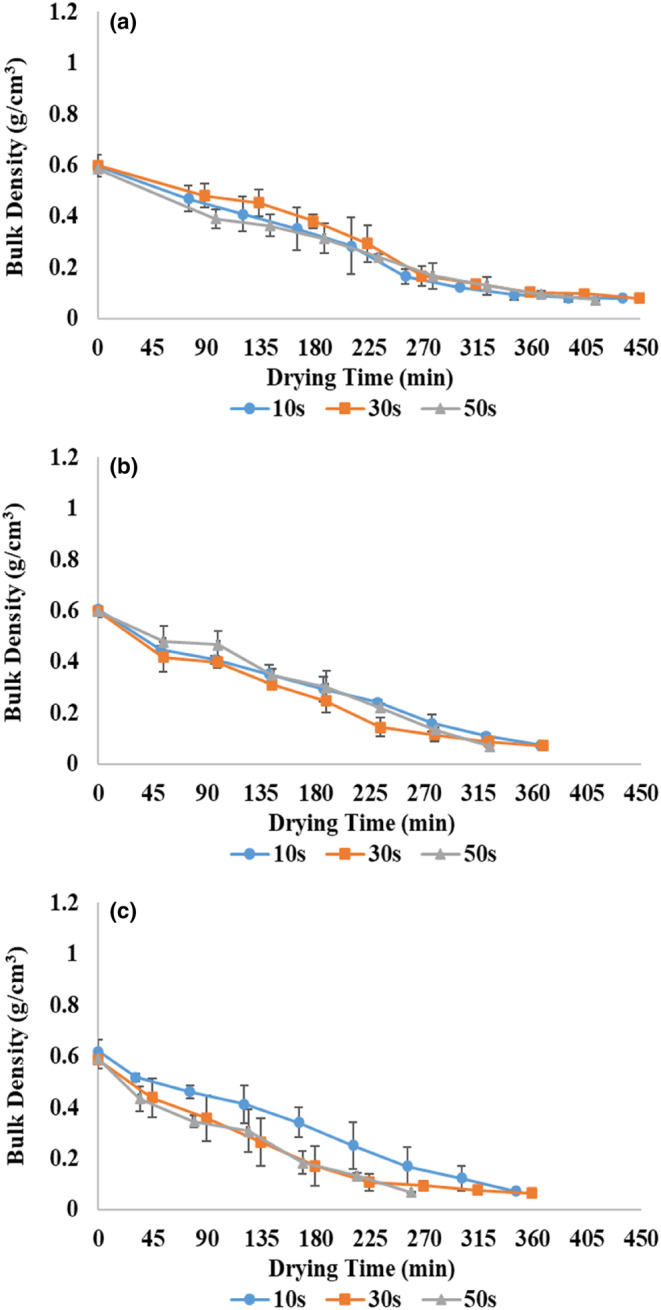
Bulk density of zucchini slices at 360 (a), 600 (b), and 900 W (c) microwave powers and application times of 10, 30, and 50 s.

Bulk density is a critical indicator for measuring the quality of food products and has a significant effect on the choice of the product by the consumer. Products with low bulk density are more acceptable due to their higher volume at constant mass because of higher porosity (Qiu et al., 2015). The change in bulk density depends on drying conditions and structural changes such as porosity and shrinkage (Dehghannya et al., 2020). Figure 9a–c shows the bulk density during the drying of zucchini using pulsed microwave–hot air at different powers and the application times. The bulk density of all treatments showed a decreasing trend until the end of the microwave drying stage (the second marker from the left) with a relatively higher slope than the hot‐air drying stage. This can be attributed to the positive effect of applying microwave energy in increasing water removal due to the high internal vapor pressure of the samples (Aghilinategh et al., 2015; Dehghannya, Bozorghi, & Heshmati, 2018). The bulk density also maintained its decreasing trend with the continuation of the drying process with hot air. Because of the progress of the process, due to the relative stabilization of the volume of the samples and the decrease in the shrinkage rate (Figure 8), the removal of moisture caused a further decline in bulk density.

In general, the bulk density decreased by 5.3% on average with the increase in application time from 10 to 50 s considering all applied powers (Table 2). This result can be related to the rise in the microwave shutdown time (Table 1), which leads to a decrease in the nonuniformity of temperature and moisture inside the sample, causing a reduction in shrinkage (Table 2) and also bulk density (Table 2; Aghilinategh et al., 2015). In addition, reducing the total time of the process (Figure 3) due to the facilitation of moisture removal by increasing the application time led to the reduction of shrinkage and bulk density.

On the other hand, the bulk density decreased considerably by 12.8% on average by increasing the power from 360 to 900 W regarding all application times (Table 2). With the increase in power, due to the substantial production of heat and the creation of a structure with more porosity due to the movement of water vapor inside the food, the bulk density decreases (Aghilinategh et al., 2015; Horuz et al., 2017; Maskan, 2000). In other words, the reduction in bulk density with increasing power was due to the decline in microwave drying time (Figure 3) and the total drying time (Figure 3), which caused a reduction in shrinkage (Table 2) too (Dehghannya et al., 2021). These results agreed with Aghilinategh et al. (2015) and Dehghannya, Bozorghi, and Heshmati (2018).

Table 2 shows the bulk density of zucchini at powers of 360, 600, and 900 W and application times of 10, 30, and 50 s. According to this table, the highest bulk density was related to the samples dried at 360 W–10 s, which their total time drying was more than other samples (Figure 3) due to the low moisture removal rate and the low *D*_eff_ (Table 2). By increasing the total drying time, the shrinkage increased (Table 2), and consequently, the bulk density also increased (Table 2). The samples dried at 900 W–30 s showed the lowest bulk density compared to other treatments, so their bulk density was 18.7% lower than the dried samples at 360 W–10 s. This improvement in quality due to the increase in power and application time can be attributed to high volumetric heating, an increase in internal water vapor pressure, the creation of more porosity as a result of reducing shrinkage (Table 2), and the reduction of drying time (Figure 3; Aghilinategh et al., 2015).

### 3.6 Rehydration ratio

**FIGURE 10:**
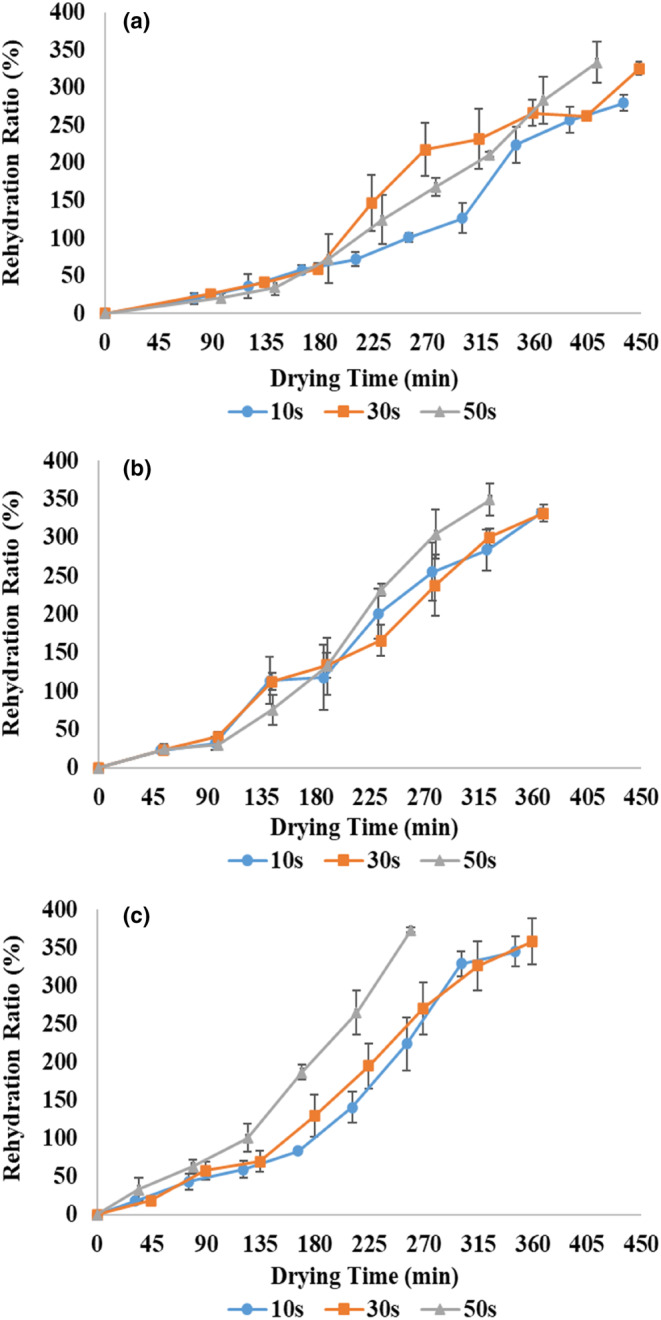
Rehydration ratio of zucchini slices at 360 (a), 600 (b), and 900 W (c) microwave powers and application times of 10, 30, and 50 s.

Rehydration indicates the intensity of tissue destruction during drying, and the higher the rehydration, the higher the quality and attractiveness of the product (Dehghannya et al., 2020; Dehghannya, Bozorghi, & Heshmati, 2018). Rehydration is a complex process affected by physicochemical alterations related to drying (Sorour & El‐Mesery, 2014). Figure 10a–c shows rehydration kinetics of dried zucchini samples during pulsed microwave–hot‐air process with powers of 360, 600, and 900 W and application times of 10, 30, and 50 s. The results showed that in all treatments, in the microwave drying stage (second marker from the left), the rehydration ratio increased. This can be related to volumetric heating and facilitating the movement of water vapor inside the sample due to the creation of more intracellular porosity in the product (Askari et al., 2006). As the process continued with hot air, the rehydration ratio increased with increasing drying time in all treatments; Because with the progress of the drying process due to the removal of more moisture, the volume and amount of water absorbed by the dried samples increased in the rehydration stage (Dehghannya et al., 2020).

In general, the results showed that the rehydration ratio increased by 10.3% on average by increasing the time of microwave application from 10 to 50 s regarding all applied powers (Table 2). As the time of application of microwave increases in fixed pulse ratio, the shutdown time also increases in the same proportion (Table 1); As a result, redistribution of heat and moisture in different places can lead to the improvement of the structure, resulting in shrinkage reduction (Table 2) and rehydration enhancement (Aghilinategh et al., 2015). Krokida and Philippopoulos (2005) also showed that reducing shrinkage leads to increased rehydration.

In addition, the rehydration ratio increased by 14.7% on average by increasing the power from 360 to 900 W considering all application times (Table 2). With the increase in the power, due to the rise in internal vapor pressure and the movement of water vapor inside the sample, cavities and non‐dense structures are created, and this decreases the total process time (Figure 3) and shrinkage (Table 2), leading to increasing rehydration capacity (Dehghannya et al., 2021; Junqueira et al., 2017; Kesbi et al., 2016). In other words, low rehydration at low powers can be ascribed to the permanent breakdown of cells, which damages the integrity of tissue and capillary tubes due to increased shrinkage (Dehghannya et al., 2021). Mounir et al. (2020) also showed that the shrinkage of the samples decreased by enhancing the power due to the reduction of the total process time, leading to the rehydration enhancement.

The results also showed that the highest rehydration was noticed in the samples that were dried at 900 W and 50 s (Table 2), in which the rehydration was 33.6% more than the samples that were dried at the power of 360 W and 10 s drying time. The drying time is increased at low powers and application times due to the decrease in the drying rate (Figure 3). As a result, it leads to the collapse and compaction of the structure, the compression of the capillary tubes, and the decrease in the hydrophilic property (Kesbi et al., 2016).

### 3.7 Specific energy consumption

**FIGURE 11:**
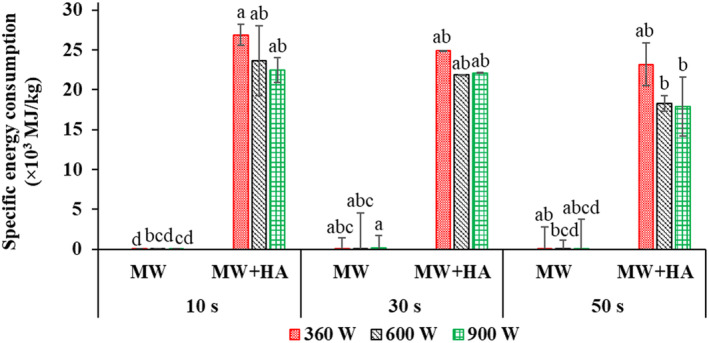
Specific energy consumption of microwave drying (MW) and total specific energy consumption [microwave and hot‐air drying (MW + HA)] of zucchini slices at microwave powers of 360, 600, and 900 W and application times of 10, 30, and 50 s. Different letters for the same drying operation (MW or MW + HA) indicate a significant difference (*p* < .05).

Figure 11 shows the energy consumed by microwave drying, hot‐air drying, and the total energy consumed in the process of zucchini drying using pulsed microwave–hot air at different powers and application times. The microwave energy consumption was significantly lower than the energy consumption of hot air due to the shorter time of microwave use (Figure 3). In other words, using microwaves in the first drying stage resulted in the removal of a certain amount of moisture (Figure 4) in a shorter time than in the hot‐air stage. The reason for this is the volumetric heating produced by microwaves and the increase in the moisture diffusion rate (Dehghannya et al., 2021). The use of pulsed microwaves can improve energy saving because the sample's heat is spread throughout it during off times. As a result, drying time is reduced by reducing the exposure time of the sample to microwaves. The energy consumed in the microwave relies on the power used and the moisture level of the product (Soysal et al., 2009).

The energy consumption substantially decreased by 18.7% on average by increasing the microwave application time from 10 to 50 s regarding all applied powers (Figure 11). By increasing the application time, *D*_eff_ increased (Table 2), and by accelerating the removal of moisture, less time (Figure 3) and energy were spent on the process (Dehghannya et al., 2021).

In addition, the results showed that the specific energy consumed considerably decreased by 16.7% on average by increasing the power considering all application times (Figure 11). Extensive heat generation with increased power increased internal water vapor pressure and, consequently, *D*_eff_ (Table 2) (Maskan, 2000). The rapid diffusion of water vapor inside the product due to the increase in *D*_eff_ improves the structure of the product and increases the porosity, accelerates the removal of moisture, and finally reduces the drying time with microwave (Figure 3), the total process time (Figure 3), and energy consumption (Figure 11) (Horuz et al., 2017). This finding was consistent with the results of Jindarat et al. (2015).

The dried treatments at the power of 900 W–50 s showed the lowest, and the dried samples at the power of 360 W–10 s showed the highest energy consumption (Figure 11). The energy consumption in the treatment of 900 W–50 s was 33.8% lower than the treatment of 360 W–10 s. The total time of the process was reduced by simultaneously increasing the microwave power and application time (Figure 3) due to the enhancement in *D*_eff_ (Table 2). As a result, the specific energy consumption was reduced.

### 3.8 Microstructure

Since the propagation of microwave to the depth of the samples causes volumetric heating and rapid moisture evaporation, significant microstructural changes occur during microwave drying. Although the evaporation rate in microwave heating is high, the product may suffer cell collapse due to the uneven distribution of heat at low pulse ratios (short shutdown times). On the other hand, long drying using the hot‐air method also causes hardening and breakage on the surface of the samples; Because in this method (unlike microwave drying), due to the low moisture diffusion rate inside the sample, shrinkage, and collapse of cells occur. Paengkanya et al. (2015) showed that the samples dried with microwave had more pore expansion (porosity) than those dried with hot air. The combined method of pulsed microwave and hot air creates a more porous structure (Pham et al., 2018).

**FIGURE 12:**
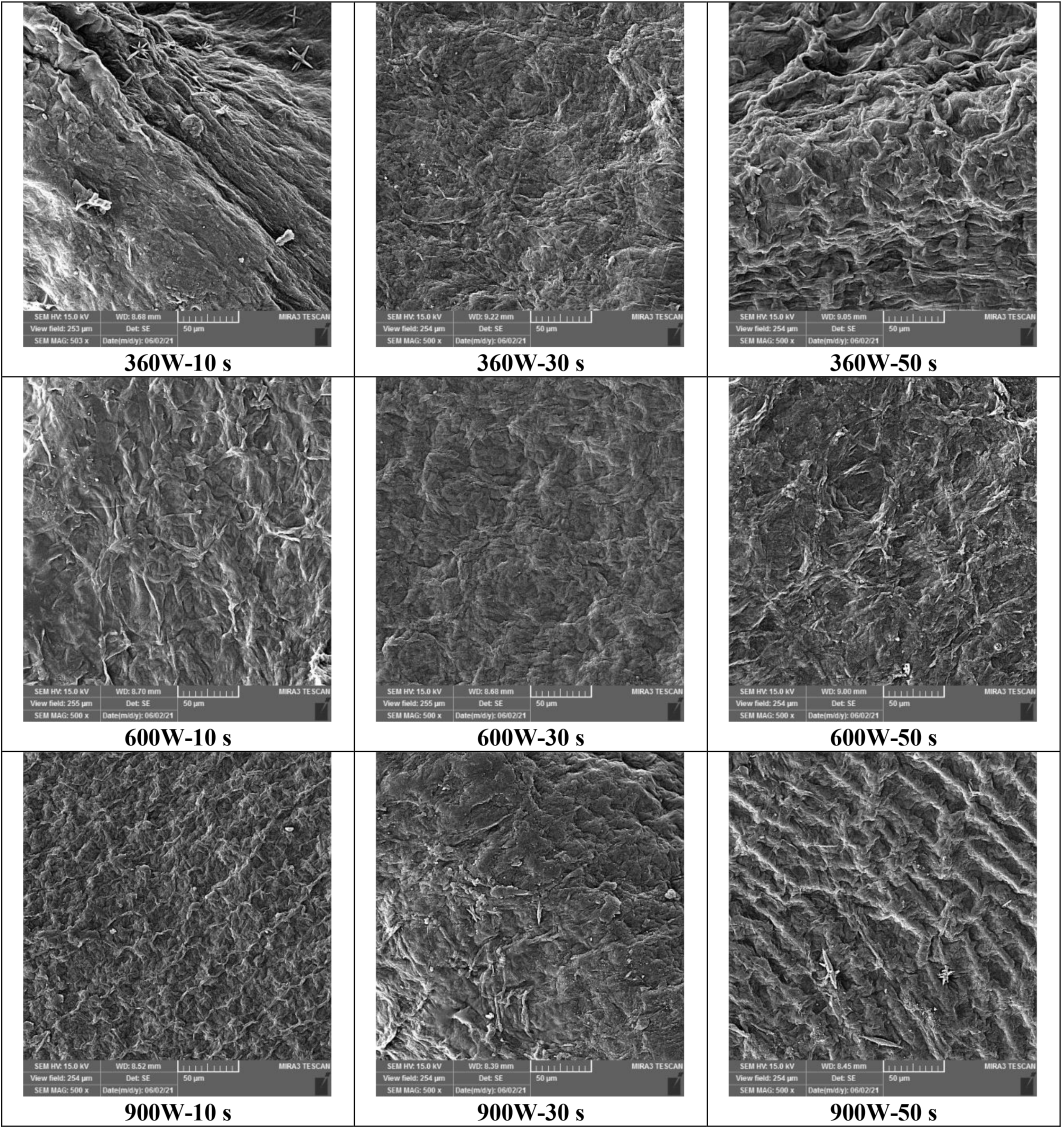
Scanning electron microscopy images of zucchini slices dried by alternating microwaves–hot air at powers of 360, 600, and 900 W and application times of 10, 30, and 50 s.

As evident in Figure 12, the number of visible pores in the sample tissue increased with increasing microwave application time from 10 to 50 s at constant power (e.g., at 360 W power). Expanding the application time at constant power leads to a rise in the internal vapor pressure inside the sample, and creates pores for the movement of water vapor. In addition, the shutdown time is increased by increasing the application time at a fixed pulse ratio (Table 1) which helps to distribute heat and moisture evenly in the sample, leading to a reduction in stress in the product (Pham et al., 2018). The increase in pores in the product structure led to a rise in the moisture removal rate (Figure 6), a decrease in bulk density (Table 2), and a reduction in the total process time (Figure 3).

According to Figure 12, the tissue shrinkage and the number of pores decreased and increased, respectively, with increasing microwave power at a constant application time (e.g., at 50 s). With the increase in power due to extensive heat and higher water vapor pressure, rapid evaporation of moisture occurs, reducing the drying time with microwaves (Figure 3) and the total drying time (Figure 3). This creates a porous structure with more pores and less collapse in the solid matrix, resulting in the reduction of bulk density (Table 2) and shrinkage (Dehghannya, Bozorghi, & Heshmati, 2018; Joardder et al., 2019; Maskan, 2000).

Considering the mutual effect of microwave power and application time, the treatment of 900 W–50 s had a more regular structure and wider pores than 360 W–10 s (Figure 12). In higher application power and time, due to the increase in evaporation rate, the total process time is reduced, and the sample is less damaged. Due to the presence of “off” times, the use of pulsed microwaves moderates the stress resulting from the rapid increase in the temperature of the sample at higher power and application time.

### 3.9 Color

#### 3.9.1 *L*, *a*, and *b*

By increasing the application time from 10 to 50 s, on average, the *L*, 9.6%, the *a*, 29.1%, and the *b*, 4.8%, showed a substantial decrease considering all applied powers (Table 3). The *L* decreases with the increase in the application time due to the rise in the temperature during the microwave “on” periods (Izli & Isik, 2015). In the presence of heat, heat‐sensitive pigments are first destroyed, which causes the formation of dark compounds and a decrease in lightness. In the next step, pigments that are resistant to heat are affected and determine the color of the product (Dehghannya et al., 2017).

**TABLE 3:** Lightness (L*), redness (a*), and yellowness (b*) of zucchini slices as influenced by different microwave application times and powers.

Microwave application time (s)	Microwave power (W)	L*	a*	b*
10	360	51.784^a^ ± 1.786	−7.644^a^ ± 2.066	54.906^a^ ± 1.553
10	600	47.160^b^ ± 1.145	−10.196^abc^ ± 0.972	52.058^ab^ ± 2.592
10	900	45.351^bc^ ± 4.412	−11.718^bc^ ± 0.390	49.518^bc^ ± 0.487
30	360	47.807^ab^ ± 1.687	−9.380^abc^ ± 0.911	49.079^bc^ ± 1.513
30	600	49.355^ab^ ± 0.701	−10.355^abc^ ± 2.146	47.740^cd^ ± 3.253
30	900	45.277^bc^ ± 2.490	−9.224^abc^ ± 2.874	48.826^bc^ ± 1.266
50	360	42.292^cd^ ± 1.214	−8.890^ab^ ± 1.824	46.774^cd^ ± 0.903
50	600	40.531^de^ ± 1.677	−11.890^bc^ ± 0.704	44.462^d^ ± 1.484
50	900	37.625^e^ ± 2.264	−12.537^c^ ± 1.897	45.127^d^ ± 1.683

*Note*: Different letters in the same column indicate a significant difference (*p* < .05).

In addition, the *L* decreased by 16.5% on average by increasing the power from 360 W to 900 W, regarding all application times, and as a result, the samples became darker (Table 3). In addition, by increasing the power from 360 W to 900 W, the *a* (redness) decreased by 12.7% on average. Also, by increasing the power, the *b* (yellowness) decreased by 12.8% averagely. These results may be related to the increased temperature of the samples at higher powers and the degradation of pigments. Similar findings have been obtained by Arikan et al. (2012), Izli and Isik (2015), Keskin et al. (2019), Arslan et al. (2020), and Keser et al. (2020).

In general, the results showed that the highest and lowest amount of L, a, and b were observed in the dried samples at 360 W power–10 s and 900 W power–50 s, respectively, so the increase in the parameters was 27.3%, 64%, and 17.8%, respectively (Table 3). Heat‐sensitive materials show more resistance in lower application power and time due to the low temperature. Since the nonenzymatic browning reaction (Maillard) is dependent on heat, as the temperature of the sample increases, darker compounds are created in the product (Soysal et al., 2009).

#### 3.9.2 Total color changes, chroma, hue angle, and browning index

By increasing the microwave application time from 10 to 50 s, the total color changes increased by 12.6% considering all applied powers (Table 4). This can be attributed to the increase in temperature during the microwave “on” time and the destruction of heat‐sensitive pigments with increasing application time. Azizpour et al. (2016) also showed that rising the temperature increased the total color changes of the dried samples. Besides, the chroma decreased with increasing application time (Table 4). This is probably related to the increase in temperature during the microwave “on” time, which leads to color degradation at higher temperatures and makes the product's color dull (Dehghannya et al., 2019; Muharrem Keskin et al., 2018). Chroma shows color saturation; In such a way that the sample's color becomes duller with its decrease and more vivid with its increase. In addition, hue angle and browning index generally decreased with increasing application time from 10 to 50 s (Table 4). This reduction can be related to a decline in the total drying time (Figure 3) and, consequently, the reduction of browning.

**TABLE 4:** Total color difference (∆*E*), chroma, hue angle, and browning index (BI) of zucchini slices as influenced by different microwave application times and powers.

Microwave application time (s)	Microwave power (W)	∆*E*	Chroma	Hue angle	BI
10	360	24.888^cd^ ± 0.663	55.464^a^ ± 1.403	−82.053^c^ ± 2.227	140.30^a^ ± 0.006
10	600	26.896^bc^ ± 2.515	53.049^ab^ ± 2.707	−78.930^bc^ ± 0.622	140.24^ab^ ± 0.116
10	900	27.682^bc^ ± 3.150	50.888^bc^ ± 0.385	−76.684^ab^ ± 0.554	140.25^ab^ ± 0.166
30	360	24.450^cd^ ± 1.942	49.972^bcd^ ± 1.588	−79.186^bc^ ± 0.879	140.34^a^ ± 0.071
30	600	23.392^d^ ± 0.788	48.905^cde^ ± 2.681	−77.639^ab^ ± 3.370	140.41^a^ ± 0.083
30	900	26.305^bcd^ ± 0.570	49.735^cd^ ± 1.781	−79.369^bc^ ± 2.958	140.28^ab^ ± 0.060
50	360	27.565^bc^ ± 1.034	47.635^cde^ ± 0.844	−79.237^bc^ ± 2.217	140.24^ab^ ± 0.083
50	600	29.619^ab^ ± 2.013	46.026^e^ ± 1.591	−75.035^a^ ± 0.512	140.26^ab^ ± 0.099
50	900	32.614^a^ ± 1.882	46.859^de^ ± 1.793	−74.485^a^ ± 2.190	140.07^b^ ± 0.211

*Note*: Different letters in the same column indicate a significant difference (*p* < .05).

In addition, the total color changes increased by 12.9% on average with increasing power from 360 to 900 W regarding all application times (Table 4). This is probably due to the increase in temperature inside the samples and its effect on the pigments of the product, and the reduction of its lightness (*L*). This finding was in agreement with Aghilinategh et al. (2015) and Sharma and Prasad (2006). Sharma and Prasad (2006) on garlic drying showed that with increasing power and temperature, the dried garlic became darker. In addition, the chroma generally decreased with increasing power from 360 to 900 W (Table 4). This decrease is probably related to the increase in the temperature of the samples with the increase in power, the decrease in the color intensity, and the increase in the opacity of the samples (Keskin et al., 2019). Also, hue angle and browning index generally decreased with increasing power from 360 to 900 W (Table 4). This is probably related to the improvement of the color of the product and the reduction of browning due to the decline in the total drying time at high powers (Figure 3).

According to Table 4, the slightest total color changes were related to the samples that were dried at 360 W power and 10 s time; Because the temperature of the product is lower in low power and time, and heat‐sensitive pigments change less. With the increase in power and temperature, nonenzymatic browning reactions of the product increase, and the resulting dark compounds also increase (Aghilinategh et al., 2015). Moreover, in a comparison between different treatments, the sample dried at 900 W and 50 s had the lowest chroma (color intensity) due to the increase in opacity at high temperatures (Muharrem Keskin et al., 2018). Also, the most downward hue angle and browning index were seen in the dried treatment with 900 W power–50 s time. This result is probably related to reducing the total process time at this power and time (Figure 3).

### 3.10 Appearance of dried samples

**FIGURE 13:**
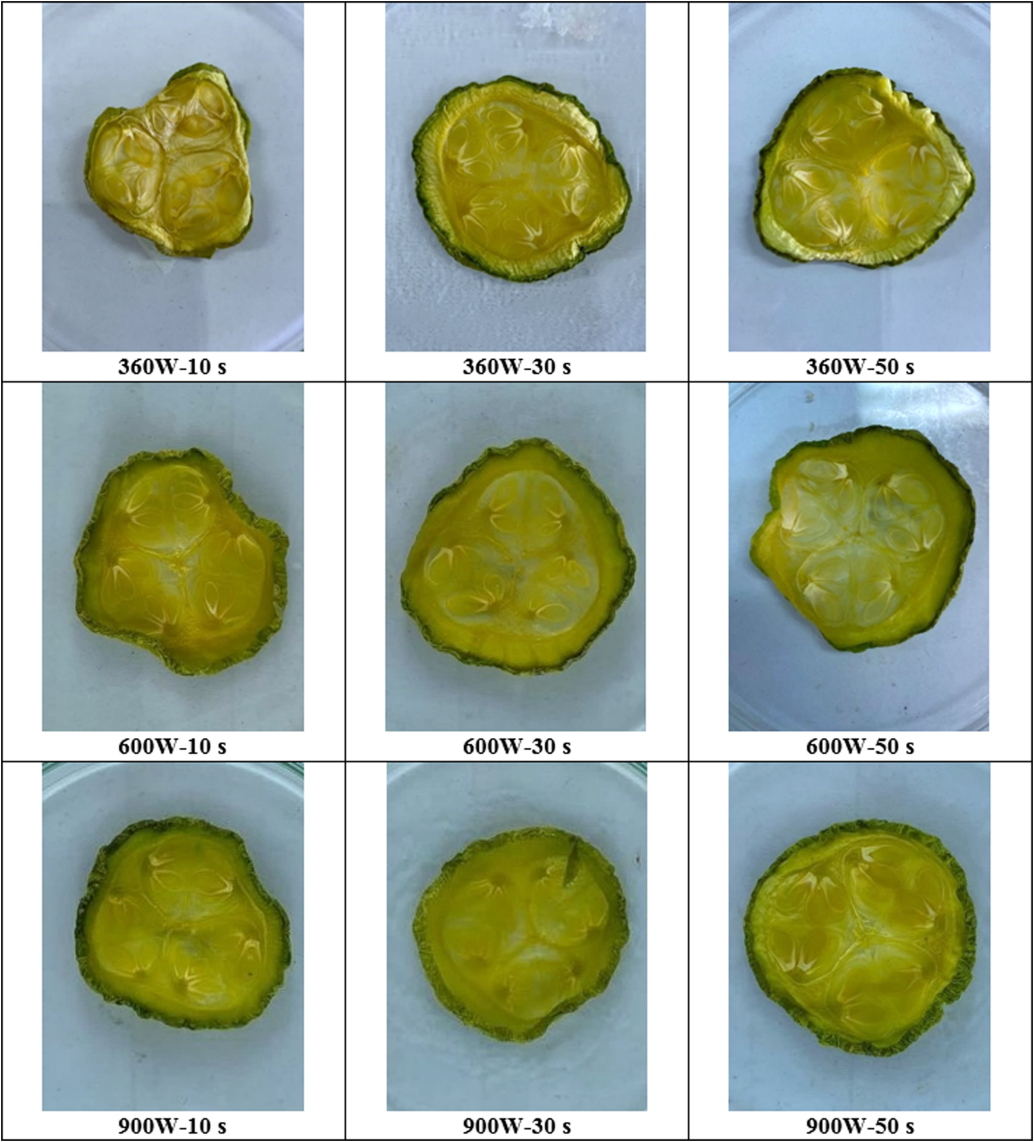
Appearance of dried zucchini slices at microwave powers of 360, 600, and 900 W and application times of 10, 30, and 50 s.

Figure 13 shows the appearance of the dried treatments at powers of 360, 600, and 900 W and application times of 10, 30, and 50 s. The highest shrinkage (the least acceptability) in appearance was observed in the dried treatment at 360 W–10 s (Table 2). This result can be related to the lengthy process time of this treatment compared to others (Figure 3). The slightest shrinkage (most acceptable) was observed in the dried sample at high power and application time (900 W–50 s). With increasing power and application time due to volumetric heating and more water vapor pressure difference, the porosity of the product increases. Due to longer shutdown time, heat and moisture distribution become more uniform, and shrinkage decreases.

## 4 CONCLUSION

The effect of pulsed microwave drying at different powers and application times was studied on the quantitative and qualitative characteristics of zucchini. Moisture removal was facilitated by increasing the power and application time due to the increase in volumetric heating and internal water vapor pressure. Besides, the more uniform distribution of heat and moisture due to more shutdown times increased the drying rate and decreased the whole process. Also, the shrinkage and bulk density decreased at higher powers and application times due to the increase in porosity. The lowest amount of rehydration and the highest energy consumption were also observed in the dried treatment at low application power and time. Increasing the power and application time due to raising the temperature of the product caused the highest total color changes. In general, by increasing the microwave power and application time (900 W–50 s), proper quality was obtained considering higher moisture removal rate, decreased shrinkage and bulk density (higher porosity), increased rehydration, and lower specific energy consumption. In conclusion, the results of this research can be used in the industry of food drying using microwaves from the point of view of controlling product quality, including reducing shrinkage, increasing rehydration, creating a porous and regular microstructure, improving color, and also reducing process time, and consequently reducing energy consumption.

## AUTHOR CONTRIBUTIONS

**Jalal Dehghannya:** Conceptualization (lead); funding acquisition (lead); project administration (lead); resources (lead); software (lead); supervision (lead); validation (lead); visualization (lead); writing – review and editing (lead). **Sepideh Farhoudi:** Data curation (lead); formal analysis (lead); investigation (lead); methodology (lead); software (lead); visualization (lead); writing – original draft (lead). **Saeed Dadashi:** Resources (supporting); visualization (supporting).

## CONFLICT OF INTEREST STATEMENT

The authors declare that there is no conflict of interest.

## Data Availability

All data generated or analyzed during this study are included in this manuscript.
